# Scrutinizing screening: a critical interpretive review of primary care provider perspectives on mammography decision-making with average-risk women

**DOI:** 10.1186/s40985-018-0092-9

**Published:** 2018-04-23

**Authors:** Sophia Siedlikowski, Carolyn Ells, Gillian Bartlett

**Affiliations:** 1 Department of Family Medicine, McGill University, 5858, chemin de la Côte-des-Neiges, Suite 300, Canada, QC H3S 1Z1 Canada; 2 Biomedical Ethics Unit, McGill University, 3647 Peel St, Room 305, Montreal, QC H3A 1X1 Canada

**Keywords:** Mammography screening, Ethics, Primary care physician, Perspectives, Decision-making

## Abstract

**Context:**

A decision to undertake screening for breast cancer often takes place within the primary care setting, but current controversies such as overdiagnosis and inconsistent screening recommendations based on evolving evidence render this a challenging process, particularly for average-risk women. Given the responsibility of primary care providers in counseling women in this decision-making process, it is important to understand their thoughts on these controversies and how they manage uncertainty in their practice.

**Objective:**

To review the perspectives and approaches of primary care providers regarding mammography decision-making with average-risk women.

**Design and methods:**

This study is a critical interpretive review of peer-review literature that reports primary care provider perspectives on mammography screening decision-making. Ovid MEDLINE®, Ovid PsycInfo, and Scopus databases were searched with dates from 2002 to 2017 using search terms related to mammography screening, uncertainty, counseling, decision-making, and primary health care providers.

**Results:**

Nine articles were included following a review process involving the three authors. Using an inductive and iterative approach, data were grouped into four thematic categories: (1) perceptions on the effectiveness of screening, screening initiation age, and screening frequency; (2) factors guiding primary care providers in the screening decision-making process, including both provider and patient-related factors, (3) uncertainty faced by primary care providers regarding guidelines and screening discussions with their patients; and (4) informed decision-making with average-risk women, including factors that facilitate and hinder this process.

**Discussion:**

The discussion of results addresses several factors about the diversity of perspectives and practices of physicians counseling average-risk women regarding breast cancer screening. This has implications for the challenge of understanding and explaining evidence, what should be shared with average-risk women considering screening, the forms of knowledge that physicians value to guide screening decision-making, and the consent process for population-based screening initiatives. Within the data, there was little attention placed on how physicians coped with uncertainty in practice. Given the dual responsibility of physicians in caring for both individuals and the larger population, further research should probe more deeply into how they balance their duties to individual patients with those to the larger population they serve.

## Background

Organized mammography screening programs have been implemented in most high-income countries since the end of the twentieth century to promote the early detection of breast cancer and reduce mortality rates from this disease. Yet, over the last two decades, the utility of these population-based programs are increasingly being questioned due to growing evidence on the uncertain benefits and potentially substantial harms of screening for average-risk asymptomatic women [1]. Since the introduction of widespread breast cancer screening in the 1980s, the incidence of invasive breast cancers has increased but the incidence of metastatic breast cancer has remained stable [2].

A decision to undertake screening for breast cancer often takes place in the primary care setting, but current controversies such as inconsistent recommendations based on available evidence on the harms and benefits of screening render this decision-making process challenging for primary care providers and their patients. One Cochrane review of 7 trials involving 600,000 women assessed the effect of breast cancer screening with mammography on mortality and morbidity [3]. It revealed that screening likely reduces mortality but the magnitude is uncertain because of methodological shortcomings of the included trials. The authors of this same review concluded that mammography screening does not clearly do more good than harm, thus underlining important ethical implications for medical practice. According to the Canadian Task Force on Preventive Health Care (CTFPHC) [4], which bases its recommendations on a systematic review of studies, regular screening only reduces the absolute risk of dying from breast cancer by 0.05, 0.13, and 0.22%, in women between the ages of 40 to 49, 50 to 69, and 70 to 74, respectively. In contrast, the United States Preventive Services Task Force (USPSTF) found higher absolute risk reductions than those of the CTFPHC. The USPSTF conducted a meta-analysis to determine the absolute rates of breast cancer mortality reduction per 10,000 women screened during a 10-year period. Their study revealed that the number of deaths reduced was 2.9 (CI, − 0.6 to 8.9) for women aged 39 to 49 years, 7.7 (CI, 1.6 to 17.2) for women aged 50 to 59 years, 21.3 (CI, 10.7 to 31.7) for those aged 60 to 69 years, and 12.5 (CI, − 17.2 to 32.1) for those aged 70 to 74 years. The absolute reduction for the combined group of women aged 50 to 69 years was 12.5 (CI, 5.9 to 19.5). Furthermore, another systematic review found that having a false positive after a mammogram could lead to lasting psychological distress [5].

Another concern is overdiagnosis, causing women to undergo unnecessary testing and treatment of cancers that would not have harmed them during their lifetime [6]. One investigation in the USA [7] found that current estimates of breast cancer overdiagnosis from screening mammography ranged from 0 to 30%. According to the authors of that study, this wide range indicates the complexity of calculating rates of overdiagnosis. They also speculate that overdiagnosis calculations may be based on studies with methodological flaws. Studies tend to use various methods to calculate overdiagnosis, and the rates measuring it differ widely. Quantifying the magnitude of the harm caused by overdiagnosis will be difficult until there is better agreement in the evidence. Despite the serious harms that some researchers have attributed to overdiagnosis using population-level data, this topic also remains challenging to assess in the context of a patient-provider relationship. In one qualitative investigation in Australia [8], researchers and policy-makers disagreed on what information should be provided to women considering screening and whether or not discussing overdiagnosis enabled or hindered informed decision-making. This study highlighted the important ethical issues around breast cancer screening communication. Another article [9] similarly discusses the challenges in communicating clinical uncertainty and the ethical problem of knowing whether communicating this uncertainty enhances or diminishes patient autonomy and offers net benefits or harms on patient experiences with care.

Basing its recommendations on the best available evidence [4, 10], in 2014, *Choosing Wisely Canad*a, a clinician and researcher-led campaign aiming to reduce unnecessary medical tests and treatments, recommended to not perform routine screening mammography for average-risk women aged 40 to 49. In contrast, the American Cancer Society [11] and The American College of Radiology [12] continue to support screening in average-risk women in this age group. The Canadian Association of Radiologists also recommend that asymptomatic average-risk women aged 40 and over should undergo screening mammography every 1 to 2 years [13].

Norris et al. studied the relationship between screening guideline panel members, their conflicts of interest, and screening recommendations for asymptomatic average-risk women aged 40 to 49 [14]. They found that five of the eight guidelines recommending screening had a radiologist member, but none of the four guidelines recommending against routine screening had a radiologist member. They also found that the proportion of primary care physicians on guidelines panels recommending non-routine screening was significantly lower than that of panels recommending routine screening.

In light of these inconsistencies in guidelines available to primary care providers, and the increasing evidence on the harms of overdiagnosis, the decision of whether or when to screen is no longer clinically or ethically obvious for average-risk women. Little is known about how primary care providers deal with these challenges in their clinical practice despite their important role in the promotion of preventive health services such as mammography screening [15]. Since primary care providers are known to influence the decision-making process of women considering screening programs [16], it is crucial to understand their perspectives regarding mammography screening and how they manage this decision-making in practice. Furthermore, codes of ethics and professional standards make clear a primary care providers’ duty to support and counsel patients in an informed consent process [17] prior to undergoing a test such as mammography screening. Thus, it is important to gain a better understanding of their views, giving consideration to ethical standards of practice.

The primary aim of our review is to explore the perspectives and approaches of primary care providers regarding mammography screening decision-making with average-risk women. Specifically in this paper, the question of what are the perspectives of primary care providers with respect to mammography screening decision-making with average-risk women will be explored.

Additionally, with respect to screening discussions with average-risk women, this review will seek information on what factors guide primary care providers in their practice and how primary care providers understand and manage clinical uncertainty, including their experiences with support of patient decision-making. To date, no review of primary care providers’ perspectives on mammography screening and decision-making with their patients has yet been published.

## Methods

A critical interpretive review of peer-review literature regarding primary care provider perspectives on mammography screening decision-making was conducted. This type of review was specifically developed for bioethics research, which typically requires the exploration of a wide range of interdisciplinary sources. The flexibility needed to conduct this review cannot function within the rigid approach of a systematic review. Instead, critical interpretive reviews offer a thorough and rigorous approach to scan literature in an effort to identify “key ideas” in a particular area of study and theorize around this knowledge, in order to answer a specific research question [18].

A search strategy was developed to identify articles capturing the perspectives of primary care providers on mammography screening recommendations and decision-making with average-risk women. In this study, the term “perspective” was broadly defined as a thought, viewpoint, or belief. Specifically, articles that examined qualitatively or quantitatively these perspectives of primary care providers about any aspect of mammography screening or mammography screening decision-making were included. Articles that discussed elements that influenced primary care providers when making screening decisions with their patients were therefore also selected. Inclusion criteria for articles were being published in English and discussing mammography screening in healthcare systems of high-income countries (Europe, North America, Australia, and New Zealand) because similar population-based screening programs have been implemented in these settings. We wished to focus the scope of this study to current perspectives based on current evidence. Since numerous mammography screening guidelines from various professional organizations and cancer societies have been published since 2002 [4, 19–21], as well as systematic reviews on harms and benefits of screening [22, 23], all articles that were published in 2002 and later were included. In the USA, family medicine, internal medicine, and obstetrics and gynecology physicians all belong to the category of primary care physicians. Since physicians in these three sub-specialties can refer women to mammography screening, articles involving any of these primary care physicians were included. We then excluded articles that exclusively discussed screening for women at a higher risk of getting breast cancer, or women outside of the 40 to 74 age range. Further, since this study aimed to capture primary care provider perspectives and approaches to screening average-risk women without a priori expectations of appropriate practice, we excluded articles measuring physicians’ adherence to mammography guidelines or those measuring their performance according to quality measures. Additionally, since this research sought to gather perspectives of primary care providers, secondary analyses of data reporting only on changes in mammography referral rates were excluded. Although it is relevant to understand the perceptions of women towards screening, since this study focused on the viewpoints of primary care providers, articles that solely presented women’s perceptions on screening were excluded. Additionally, articles reporting the perspectives of professionals in medical specialties other than primary care such as radiology were excluded, because they do not operate in a preventive medicine context. Viewpoints stemming from empirical evidence were prioritized over those arising from anecdotal evidence. Although critical analyses, editorials, and commentaries from primary care providers were included in order to scan for relevant references of empirical data on the perceptions of primary care providers, no new references were obtained this way; thus, these articles were ultimately excluded.

## Search strategies

The databases Ovid MEDLINE(R), PsycInfo, and Scopus were scanned from 2002 to 2017 on 23 February 2018 using categories of search terms relating to mammography screening, counseling, decision-making, overdiagnosis, consent, and those covering primary health care provider terms. All combinations of terms were covered, and mappings to headings were made wherever possible. The Cochrane database was also scanned for potential relevant articles, but this search did not identify any eligible papers. The specific search strategies for the three databases were as follows:

- Ovid MEDLINE(R) search 2002–present: Mammography/ or mammogr* or breast cancer screening AND mass screening or early detection of cancer; OR screen*; AND Counseling or counsel* or (overdiagnos* or over diagnos*) or practice patterns, physicians’/ or decision-making or decid* or informed decision-making or informed consent or consent* or Uncertainty/ or uncertain* AND (family or physician$).af. or practice$.mp. or primary care.af. or exp. Primary Health Care/ or primary.mp. or general pract$.af. or gp.tw. or gps.tw. or nurses/ or nursing/ or nurs*.
- Ovid PsycInfo search 2002–present: Mammography/ or mammogr* or breast cancer screening AND Cancer screening or screening or screen* AND Counseling or counsel* or (overdiagnos* or over diagnos*) or decision-making/ or decid* or informed decision-making or informed consent/ or consent* or Uncertainty/ or uncertain* AND (family or physician$).af. or practice$.mp. or primary care.af. or exp. Primary Health Care/ or primary.mp. or general pract$.af. or gp.tw. or gps.tw. or nurses/ or nursing/ or nurs*.
- Scopus search 2002–present: TITLE-ABS-KEY(mammogr* OR “breast cancer”) AND TITLE-ABS-KEY(screen*) AND TITLE-ABS-KEY
  (counsel* OR decid* OR decision* OR uncertain* OR consent* OR overdiagnosis) AND TITLE-ABS-KEY(“family physician” OR “family doctor” OR “primary care*” OR “primary health*” OR “general pract*” OR nurse OR “nurse pract*”).

Following an in-depth reading of the results sections of all included articles, data were organized into sections that sub-divided the main objective of the review. The thematic development in critical interpretive reviews requires an inductive and iterative analytical approach. Through this process, the analysis was revised and refined until all relevant elements from the articles were appropriately captured into three final sections. Drawing on Sally Thorne’s interpretive descriptive framework [24], content from the included articles were then interrogated against professional and ethical codes of practice such as the Canadian Medical Association Code of Ethics [17] and Code of ethics of physicians in Quebec [25].

## Results

The database searches resulted in 1423 articles. After removal of duplicates, the three database searches yielded a total of 761 articles. One team member (SS) then reviewed the titles and abstracts of these articles and retained those that seemed to address the aim of this study. This search strategy identified 50 articles. Two team members (SS and CE) then independently reviewed these 50 articles in more depth and met in person to discuss which of these should be included based on our criteria. We also reviewed the reference lists of these retained articles to identify any other relevant articles that were not captured through our database searches. When needed, a third team member (GB) was consulted to reach consensus on whether an article should be included. Following this process, we identified a total of nine empirical studies [26–34].

Since all participants in the included articles were physicians working in primary care, for simplicity, we report the results and follow using the term “primary care physicians” (PCP) and in some places “physicians”. The analysis of data in the nine articles resulted in a grouping of results into four thematic categories. The first grouping includes general clinical perspectives and approaches from physicians on screening such as their perceptions on the effectiveness of screening and at what age they initiated screening with average-risk women. The second group includes data on the multiple factors guiding physicians in the screening decision-making process. This category was the richest in data, and findings touched on physician- and patient-related factors, the influence of best practice guidelines and physicians’ sub-specialty organizations, as well as non-medical factors such as physicians’ colleagues’ influence on their practice. The third category of results reports on data relating to the uncertainty faced by physicians with regard to guidelines and screening discussions with their patients. The last thematic grouping includes all data discussing decision-making approaches. Physicians’ thoughts on their willingness to support women in informed decision-making and the factors facilitating and hindering the informed decision-making for average-risk women are presented.

Table 1 presents the key characteristics of the included articles. Although all included articles are empirical, a variety of different outcomes were assessed. Authors measured the initiation and frequency of screening, the decision to order screening, the level of agreement of PCPs with different guidelines and if they were perceived as unclear, the influence of guidelines and non-medical factors in the decision to recommend screening or not, and the perceived effectiveness of mammography in reducing breast cancer mortality. They additionally measured the perceptions of physicians on patient anxiety and patient needs. Most articles used surveys to collect data quantitatively but one article [32] qualitatively explored the experience of physicians counseling patients and patients’ views on this decision-making process using interviews and focus groups. Data in these nine articles were collected between 1999 and 2016 in Canada or the USA.

**Table 1:** Key characteristics of included articles

Article^a^	Objective	Setting	Year and method of data collection	Relevant outcome measures^b^
Tudiver 2002	To determine perceptions of family physicians on unclear or conflicting guidelines including mammography for women aged 40–49, and what factors influence their decision to order these tests	Canada	1999, National mailed survey with case vignettes	Agreement with guideline statements; decision to order screening test; factors that influence this decision
Haggerty 2005	To compare the influence of family physicians’ recommendations and patients’ anxiety or expectations on the decision to order screening tests for which guidelines are conflicting, including mammography for women 40 to 49	Canada	1999, Secondary analysis of the survey from Tudiver [26] with clinical case vignettes	Decision to order screening test; perceptions of mammography recommendations; physician perception of patients’ anxiety about cancer expectations to be tested
Meissner 2011	To explore the mammography screening beliefs, recommendations, and practices of primary care physicians in family medicine, general practice, internal medicine, and obstetrics/gynecology, for average-risk women aged 40–49 and over 50	USA	September 2006 to May 2007, Nationally representative survey of PCP	Influence of guidelines in clinical practice of PCP; beliefs about the effectiveness of 4 breast cancer screening tests in reducing breast cancer mortality in average-risk women; mammography recommendations to asymptomatic average-risk women; recommended frequency of mammography for women aged 40–49 years and aged > 50 years; age at which PCP no longer recommended screening for healthy women
Smith 2012	To determine family medicine residents’ fellows’ and staff physicians’ attitudes and behaviors towards breast cancer screening in average-risk women aged 40 to 49	Canada, Two academic family practice health centers	No date reported^e^, Cross-sectional survey	Screening initiation and frequency; reasons for offering and not offering screening; physicians’ perceptions of patients’ needs and understanding regarding mammography screening
Miller 2014	To examine family medicine, internal medicine, and obstetrics and gynecology physicians’ beliefs about the effectiveness of different tests for cancer screening in women 40 to 49 and 50–69	USA, Private practice and hospital	November 2008 to January 2009, survey with data from *Women’s Health Survey* sent to a nationally representative sample of physicians	Level of agreement with statements that tests were effective in screening for breast cancer; professional organizations influencing physicians’ cancer screening recommendations
Kiyang 2015	To assess the intention of family physicians to support women aged 50 to 69 (targeted by the QBCSP^c^) in making informed decisions about mammography, the determinants of this intention, and the factors that influence family physicians’ adoption of this supporting behavior	Canada	2010, Questionnaire based on the Theory of Planned Behaviour post-attendance to a lecture on informed decision-making	Physicians’ intentions to support women in making informed decisions about mammography screening; determinants of this intention and the barriers and facilitators to adopting this supportive attitude.
DuBenske 2017	To compare women’s and primary care physicians’ (Family medicine, Internal medicine, obstetrics and gynecology) experiences of mammography screening shared decision-making with average-risk women aged 40 to 49.	USA, Academic health center and clinics	2013, Patient focus groups with women aged 40 to 49 and interviews with primary care physicians	Primary Care Physicians’ and patients’ experiences in mammography screening decision-making
Radhrakrishnan 2017	To assess the associations between screening recommendations and (1) physician specialty and (2) organizational trust	USA	2016, National survey of primary care physicians	Physicians’ screening recommendations; physicians’ most trusted screening guidelines
Radhakrishnan 2018^d^	To investigate a broad range of attitudes and beliefs towards mammography screening, using factor analysis to group them into underlying themes	USA	2016, National survey of primary care physicians	Physician attitudes towards mammography screening for younger (45–49 years) and older (75+ years) women; recommendations for routine mammography

^a^First author and year of publication

^b^Only the outcome measures relevant to the aims of this critical review are provided in this table

^c^Quebec Breast Cancer Screening Program

^d^Only data concerning the younger group of women aged 45–49 were considered in this review

^e^No date reported in article. The first author was contacted by email October 26, 2017, but no reply was received by date of submission to the journal

Table 2 summarizes the mammography screening recommendations of organizations cited in the included articles. Since the studies report on the perspectives of PCPs from 1999 to 2013, this table is shown to highlight the guidelines that were available to the participants in the included studies at the time of data collection.

**Table 2:** Summary of mammography screening recommendations in effect during data collection periods for the included articles

Guideline	Mammography screening recommendations for average-risk women^a^		
	Aged 40 to 49	Aged 50 to 69	Aged 70 to 74
Canadian Task Force on Preventive Health Care [4, 43]	2011: no routine screening (weak recommendation; moderate quality evidence)	2011: routine screening every 2 to 3 years (weak recommendation; moderate quality evidence)	2011: routine screening every 2 to 3 years (weak recommendation; low quality evidence)
	2001: no recommendation (grade C). Screening should be an individual’s decision	2001: routine screening every 1 to 2 years	2001: routine screening every 1 to 2 years
United States Preventive Services Task Force [19, 21, 44]	2016^b^: the decision to start screening mammography in women prior to age 50 years should be an individual one. Women who place a higher value on the potential benefit than the potential harms may choose to begin biennial screening between the ages of 40 and 49 years (grade C)	2016^b^: biennial screening (grade B)	2016^b^: biennial screening (grade B)
	2009: the decision to start biennial screening before age 50 should be an individual one and take patient context into account, including the patient’s values regarding specific benefits and harms (grade C)	2009: biennial screening (grade B)	2009: biennial screening (grade B)
	2002: screening every 1 to 2 years (grade B)	2002: screening every 1 to 2 years (grade B)	2002: screening every 1 to 2 years (grade B)
American Cancer Society [11]	Since 2003: women should begin annual mammography at age 45 and should be able to start at age 40 if they would like		
American Congress of Obstetricians and Gynecologists [45]	Since 2003: annual mammography screening should be offered to women 40 years and older		
American Academy of Family Physicians [46] and American College of Physicians [47]	After 2009: biennial screening for women aged 50 to 74 years		
	Before 2009: screening starting at age 40 every 1 to 2 years		

^a^When reported, the rating for the quality of the evidence is listed with the GRADE score [4, 48]

^b^Guidelines that have been updated since the included studies’ publications have been listed [21]

In two articles [28, 30], physician participants were asked to rate the influence of the USPSTF guidelines, and in two others [33, 34], these physicians rated their level of trust in different organizations including the USPSTF. Physicians were also asked about the CTFPHC in two articles [26, 27], and this guideline was cited in one other article [29]. Moreover, the five American studies [28, 30, 32–34] made reference to PCPs’ sub-specialties’ guidelines, so mammography recommendations for the American College of Obstetricians and Gynecologists (ACOC), the American Academy of Family Physicians (AAFP), and the American College of Physicians (ACP) are listed. The two North American task force organizations currently recommend routine screening in average-risk women between the ages of 50 and 74. For women in the 40 to 49 age range, the CTFPHC recommends against screening since 2011 and as of 2009, the USPSTF gives no recommendation and views screening as an individual’s decision. The AAFP’s recommendations align with the USPSTF’s 2016 updated guideline, but ACOG still recommends that screening should be provided to women starting at age 40. The ACP recommends that screening starts at 45, an earlier screening starting age than the AAFP’s and USPSTF’s starting age of 50.

Data reporting on the general perspectives and approaches of PCPs regarding mammography screening for average-risk women are shown in Table 3.

**Table 3:** Primary care physician beliefs on screening effectiveness and practice behaviors

Article	
Tudiver 2002	NA^a^
Haggerty 2005	• Approximately 25% of the participating physicians thought that routine mammography screening was recommended for women aged 40–49 years.
Meissner 2011	• 99% of all PCPs reported that for average-risk women 50 years and older, mammography was effective in reducing cancer mortality.
	• 96% thought that mammography was at least somewhat effective for women ages 40 to 49 years.
	• Over 70% of all physicians who recommended mammography to women ages 40 to 49 years recommended it on an annual basis (69.5% of family medicine/general practitioners, 74.5% of internal medicine specialists, and 79.3% of obstetrician/gynecologists).
	• More than 90% of all physicians recommended annual mammography to women aged > 50 years. Family medicine/general practitioners and internal medicine specialists who recommended mammography were more likely to stop recommending screening at a certain age (30.2 and 37.8%, respectively) than obstetrician/gynecologists (14%).
	• The age at which MDs no longer recommended screening varied, but less than 10% of physicians of any specialty specified an age that was smaller than 70 years.
Smith 2012	• 46% of family physicians offered routine mammography screening to average-risk women aged 40–49.
	• Among physicians who offered screening: 77% reported starting at age 40, while 14% started at age 45. Of these, 44% offered yearly screening, followed by 26% who offered biennial screening. The remainder of physicians offered either annual or biennial screening based on joint physician-patient decisions (17%).
Miller 2014	• 50% of physicians strongly agreed that mammography is an effective test for women aged 40–49 years.
	• 81.7% of physicians strongly agreed that mammography is an effective screening test for women aged 50–69 years.
Kiyang 2015	NA^a^
DuBenske 2017	NA^a^
Radhakrishnan 2017	• 81% of physicians recommended screening to women aged 40 to 44 years.
	• Gynecologists were more likely than family medicine/internal medicine physicians to recommend screening for women in general.
Radhakrishnan 2018	• 88% of physicians recommended screening mammography to women aged 45–49 years.
	• Of those physicians, approximately 67% recommended yearly screening for that group of women.

^a^NA, not applicable

The participating physicians in three articles found mammography guidelines unclear or conflicting [26, 27, 32]. At least 45% of the participating physicians in two studies [28, 29] routinely recommended and offered screening to women between the ages of 40 and 49. In another study [27], a smaller proportion of physicians, less than 30%, thought that routine mammography was recommended for women in this age range.

Table 4 presents the various factors guiding PCPs with respect to mammography screening decision-making with average-risk women.

**Table 4:** Factors guiding primary care physicians in the decision-making process regarding mammography screening with average-risk women

Article	
Tudiver 2002	• Patient anxiety, patient expectations of being tested, and a positive family history of breast cancer all significantly increased the chances that a mammogram would be ordered.
	• MDs’ beliefs that mammography was not recommended or causes more harm than good, and a good patient-doctor relationship decreased the odds of screening.
	• The sensitivity of MDs to their colleagues’ practice increased the odds of screening.
Haggerty 2005	• The physicians who believed routine screening was recommended ordered the test in most cases regardless of patient characteristics.
	• Physician beliefs about screening strongly predicted test ordering, but only when patients had no anxiety or expectations. If a physician thought that mammography for women aged 40 to 49 was not recommended or was unclear, then a patient’s expectation of having mammography tripled the probability that mammography would be ordered.
	• If a physician perceived that routine mammography was recommended, however, then a patient’s expectation did not alter significantly the already high likelihood that a physician would order the mammography test.
	• Family physicians agreed that numerous non-medical factors influenced their usual test-ordering behavior.
	• 89.6% of physicians stated they would order a screening test that they would not usually recommend if the specialists with whom they work recommended the test
	• 88.1% would order the test if a patient requested the test and insisted on having it done.
	• 87% would order it if a patient was anxious about having the disease.
	• 59.2, 57.2, and 54.7% of physicians would order the test if it was easy to administer, easily accessible, and inexpensive, respectively.
	• If their colleagues were recommending the test to their own patients, 37% of physicians said they would order the test.
	• Approximately 30% of physicians said they would order the test if it would take less time than convincing patients that they do not need it.
Meissner 2011	• Most physicians identified at least 1 breast cancer screening guideline as being very influential in their practice.
	• The ACS guidelines were most frequently cited as influential (56%), followed by the ACOG (47%), USPSTF (42%), AAFP (32%), and ACP (25%) guidelines.
Smith 2012	• 40% of physicians did not think breast cancer screening was necessary for women aged 40 to 49, but 62% said they would order the test if their patients requested it.
	• Reasons to not offer screening:
	- No evidence of decreasing breast cancer related deaths (63%)
	- Grade A recommendation to screening at age 50 and not 40 (25%)
	- Harms of screening outweighing benefits (19%)
	• Reasons to offer screening:
	- Patient request (55%)
	- Personal practice or mentor recommendation (27%)
	- Guideline recommendation (18%)
	- Other reasons to offer screening included emerging evidence of a modest decrease in breast cancer mortality, detection of early-stage breast cancer, and improvement in imaging for detecting benign versus malignant lumps.
Miller 2014	• The majority of physicians ranked their respective specialty professional organization as one of the top organizations that influenced their cancer screening recommendations.
	• Across all three specialties, the majority of physicians reported the ACS as a top influential organization.
	• More than 50% of Family Medicine and Internal Medicine physicians reported the USPSTF, as their top influential organizations.
	• Almost 50% of the Obstetrics and Gynecology physicians ranked the National Institutes of Health/National Cancer Institute as one of their top influential organizations.
	• Physicians who listed the ACS as one of their top influential organization were significantly more likely to believe that mammography was effective for women 40–49.
	• In contrast, physicians who listed the USPSTF as their top influential guideline were less likely to believe that mammography was effective for women age 40–49.
	• Physicians who reported a personal cancer experience were less likely to believe that mammography is effective for women aged 50–69 years.
Kiyang 2015	NA^a^
DuBenske 2017	• Physicians report concerns for time constraints and desire for efficiency in decision-making discussions.
	• Women identify the need for physicians to take time to listen to their concerns and answer questions (reported as a discordance with the finding from the physician interviews).
Radhakrishnan 2017	• Physicians who trusted ACS and ACOG were significantly more likely to recommend screening to younger women compared with those who trusted USPSTF guidelines.
Radhakrishnan 2018	• 26% of physicians trusted ACOG guidelines the most, 23.7% ACS, and 22.9% UPSTF.
	• The most trusted guidelines for gynecologists, family medicine/general practitioners, and internists were respectively those by ACOG, USPSTF, and ACS.
	• Factors leading to physicians recommending screening:
	(1) Physicians had feelings of potential regret from not ordering mammograms:
	- Higher risk for malpractice liability
	- Fear or missing potentially lethal cancels
	- Patient’s expectations about mammograms
	(2) Concerns with and leading to overuse of screening

^a^NA, not applicable

Three of the included studies [28–30] collected data on the influence of practice guidelines on physicians’ ordering of mammography screening. In two of the American studies [28, 30], the American Cancer Society was identified as the most influential screening guideline.

One of these studies, however, showed that PCPs in the USA were most influenced by their sub-specialty cancer screening guidelines [30]. In one other American study, physicians who trusted the USPSTF the most were significantly less likely to recommend mammography screening to women aged 40–49 than those who most trusted other organizations [33].

Furthermore, three of the studies revealed that physicians would recommend screening if their colleagues recommended this test [26, 27, 29]. As many as 89.6% of physicians in one study [27] stated they would order a screening test that they would not usually recommend if the specialists with whom they worked recommended the test. In addition, patient anxiety about having cancer and patient expectations to have mammography increased the likelihood that a physician would order a screening test [26, 27, 29, 34]. In one particular case [29], 40% of physicians did not think breast cancer screening was necessary for women aged 40 to 49, but 62% of those physicians said they would order the test if their patients requested it. Of the physicians who did not offer screening to women 40 to 49 [29], the most commonly expressed reason for not screening was the absence of evidence of decreasing breast cancer-related deaths with screening. In the same study, approximately 20% of physicians in that study said they did not offer mammography screening because they thought the risk of harms such as increased anxiety, unnecessary radiation exposure, high false positive rates, unnecessary biopsies, and overtreatment of benign results outweighed any benefits of the screening. In a second article [27], if a physician thought that mammography for women aged 40 to 49 was not recommended or was unclear, then a patient’s expectation of having mammography tripled the probability that mammography would be ordered. Only one article [26] reported on the patient-doctor relationship as a factor influencing a physician’s decision to order a screening test. In this study, a good quality patient-doctor relationship significantly decreased the odds that physicians would order mammography screening for women aged 40 to 49.

Three of the articles [26, 27, 32] reported on uncertainty in the area of mammography screening and these data are shown in Table 5.

**Table 5:** Primary care physician perspectives on uncertainty in mammography screening

Article	
Tudiver 2002	• Over 65% of physicians found mammography screening guidelines conflicting.
Haggerty 2005	• About 30% of physicians found mammography screening guidelines unclear.
Meissner 2011	NA^a^
Smith 2012	NA^a^
Miller 2014	NA^a^
Kiyang 2015	NA^a^
DuBenske 2017	• Physicians are not always aware of all risk factors or using all risk factors in their discussions.
	• Physicians identified ambiguity in the guidelines.
	• Physicians reported less confidence in their ability to know or consider all risk factors for an individual’s risk calculation as well as difficulty making sense of ambiguous, contradictory or changing guidelines.
	• One physician stated he did not feel adept to discuss screening.
Radhakrishnan 2017	NA^a^
Radhakrishnan 2018	• The difficulty of reconciling divergent organizational guidelines was strongly associated with recommending screening to women aged 45–49.
	• Physicians who trusted the USPSTF guidelines the most had lower potential regret.

^a^NA, not applicable

In these studies, physicians found mammography guidelines unclear, contradictory, and changing. One study showed that the difficulty of reconciling divergent organizational guidelines was strongly associated with recommending screening to women aged 45–49 [34]. The study involving interviews with physicians [32] revealed that physicians did not feel confidently prepared to have a discussion with their patients about mammography screening and struggled with this uncertainty.

Lastly, the physicians’ perspectives on the mammography decision-making process between physicians and patients are found in Table 6.

**Table 6:** The decision-making process about mammography screening including influencing factors

Article	
Tudiver 2002	NA^a^
Haggerty 2005	• Approximately 30% of physicians said they would order the test if it would take less time than convincing patients that they do not need it.
Meissner 2011	NA^a^
Smith 2012	• 94% of physicians found patients often or always thought that breast cancer was a serious threat, were aware of screening and wanted to discuss screening mammography.
	• Overall approximately 75% of physicians said that lack of time was never or rarely an issue in discussing breast cancer screening with patients aged 40–49.
	• 55% of physicians said they discussed the risks and benefits of screening with their patients, and allowed them to decide when screening mammography should be initiated.
Miller 2014	NA^a^
Kiyang 2015	• 63% of MDs showed strong or very strong intentions to support women in making informed breast cancer screening decisions.
	• Perceived behavioral control was most strongly associated with intention to support, followed by attitude, and then social normal.
	• Physicians most frequently reported time constraints as a barrier to supporting women, followed by women’s awareness of relevant information.
	• The most frequently reported facilitator of supporting women was the availability of decision support tools for physicians and their patients.
	• The next most reported facilitators were specific characteristics of targeted women and the physicians’ own knowledge about informed decision-making.
DuBenske 2017	• Physicians reported struggling to discuss screening mammography.
	• Four elements had a critical impact on communication between family physicians with patients on the shared decision-making process: (a) Time constraints; (b) Risk (lack of adequate knowledge of risks and ability to communicate risk in an effective format); (c) Guidelines (confusion related to conflicting and changing guidelines); and (d) personal preferences (addressing patient preferences that contradict guidelines and addressing physician’s own biases).
	• Physicians reported a concern for time constraints, and noted they act as a barrier on being able to thoroughly consider all risk factors and offer individual recommendations. They also desired efficiency in the screening discussion.
	• Physicians report that they do have brief conversations about potential outcomes of screening, yet women in this study reported receiving limited or no information about them.
	• Both identify and support patient preference for varying degrees of involvement in decision-making. Both desire women to understand their risks. Both see the value in preparing women for potential call-backs and next steps, however, women report this does not happen whereas many physicians reported that they do discuss this.
	• Many women trust their physicians understand guidelines and use them in directing their decision; physicians identify ambiguity in the available guidelines.
Radhakrishnan 2017	NA^a^
Radhakrishnan 2018	NA^a^

^a^NA, not applicable

Four of the articles highlighted time as a factor affecting the screening decision-making process [27, 29, 31, 32]. In two of these studies [31, 32], physicians reported lack of time as a barrier to supporting women making informed decisions and a desire for efficient discussions. Approximately 30% of the physicians in a third study [27] stated they would order mammography if it would take less time than convincing patients that they do not need it. In contrast, the majority of the physicians in a fourth study [29] said that time was never or rarely an issue in mammography screening discussions. An overwhelming proportion of the physicians in this same study also perceived that women wanted to discuss screening mammography, yet only 50% of the physicians claimed to discuss the risks and benefits of screening with their patients.

## Discussion

The reviewed literature offers an overview of the current mammography screening landscape from the perspective of PCPs. These physicians approach mammography screening with average-risk women in different ways and hold diverse views with respect to screening decision-making with their patients, based on differing beliefs and varying factors influencing their practice. This research is useful to further understand what guides physicians when clinical guidelines are unclear and conflicting and sheds light on the extent to which other factors consequently play a role in decision-making. By narrowing in on the patient-PCP relationship, this research illustrates what actually occurs in physicians’ offices, regardless of public health messages or population-based mammography program goals. It can inform next steps on identifying what physicians need to improve the mammography screening decision-making process with average-risk women in order to respect ethical and professional obligations towards their patients.

The PCP data revealed that more than 50% of physicians in three of the nine included studies found mammography guidelines unclear, conflicting, or ambiguous. We expected studies to report on this clinical uncertainty in the recommendations, but interestingly, the physician data did not extensively elaborate on the ways in which physicians coped with clinical uncertainty in mammography decisions. Only one article [32] revealed that physicians reported less confidence in their capabilities of engaging in screening discussions with patients due to ambiguous guidelines. We had also anticipated capturing some data on ethical tensions experienced by physicians due to this lack of clarity in practice guidelines and to controversies about overdiagnosis. These tensions could include the willingness to justly inform women about the benefits and risks of mammography screening without causing undue distress by discussing screening drawbacks such as overdiagnosis, and uncertainties around the magnitude of this problem. Tensions between ethical principles in the decision-making process may not have come through our search because we did not include the keyword ethics. Or, the absence of data on ethical tensions could be due to a low likelihood of empirical studies measuring outcomes related to ethical or moral tensions.

Data from two studies [28, 30] showed that physicians clearly believed in the effectiveness of mammography screening in reducing breast cancer mortality, despite evidence in a systematic review showing the limited effectiveness of this screening test [4]. Since the effectiveness of mammography in preventing death from breast cancer and the rates of false positives and overdiagnosis vary by age group, sharing these numbers with women might improve screening discussions between providers and patients [35]. Despite this variability in screening effectiveness across age groups, the numbers of women needed to be screened in order to prevent one death from breast cancer remain substantial. In one systematic review [4], the authors conclude that 2108 and 721 women would need to be screened every 2 years for a median of 11 years in order to prevent one death from breast cancer in women between the ages of 40–49 and 50–69 years, respectively. Yet, despite this low absolute risk reduction associated with regular screening for average-risk women, up to 50% of the physicians in one study [28] strongly agreed that mammography is an effective test for women aged 40 to 49. Some PCPs may hold this belief because they are unaware of evidence on numbers needed to screen, or because of a misunderstanding in the evidence they access to inform their clinical practice. In their work on the ethics of screening [36], Juth and Munthe note that some researchers express reductions in mortality from breast cancer using relative risk, while expressing rates of overdiagnosis and overscreening with absolute risk. They point out that presenting data in this way is conducive to biases favoring screening by “playing down the negative effects and emphasizing the positive ones”. Framing the benefits and harms of screening tests such as mammography using different types of risk may be confusing to clinicians and work against efforts to promote informed consent and patient autonomy.

PCPs have discussed this issue of presenting evidence on risk reductions associated with tests or interventions as absolute versus relative risk and how this difference has an impact on the capability of women to make informed choices. Woloshin and Schwartz [37] affirm that in a world where selling screening is much easier than selling informed choice, women needed to be reminded that “screening is a genuine choice”. These physicians acknowledge the disagreement in the evidence despite the substantial amount of research that has been conducted on mammography harms and benefits. In counseling patients, they propose using screening fact tables that convey as clearly the possible the order of magnitude of the effects of regular mammography screening.

Yet, even if providers are equipped with the necessary information to share with women considering screening, disagreements on what exactly should be shared with women remains a problem. For instance, in Parker et al.’s work on breast cancer screening communication [8], the most frequently expressed reasons for the provision of information on overdiagnosis included: the right for people to know what they are signing up for when they participate in screening and the idea that providing information enables informed decision-making which is particularly important for breast cancer screening given the drawbacks. In contrast, the most commonly expressed rationale to limit information on overdiagnosis was that doing so maximized screening participation. The participants in this study, however, were not asked about their beliefs regarding screening effectiveness in reducing breast cancer mortality, which would be important to look at if they hold the belief that maximizing screening is important. The participants who advocated for limiting information on overdiagnosis challenged the concept of overdiagnosis as a harm. They thought that increased participation in screening would enhance patient choice later on, given the importance of early detection of breast cancer in treatment decisions.

Regardless of what specific information is presented to patients, using evidence to guide practice requires proper knowledge and understanding of statistics on the provider’s part to distinguish between relevant and irrelevant data. In a survey of over 400 PCPs in the USA [38], nearly half of the physicians mistakenly thought that a higher incidence of cancer in a screened population versus an unscreened group meant that the screening test saved lives. Although this data on statistics illiteracy is limited by the authors’ use of hypothetical scenarios, these findings are concerning. Providers are expected to practice medicine according to evidence and should be able to explain their reasoning behind recommending a test or not, by understanding the numbers supporting their stance. Yet, even with a thorough understanding of statistics, the findings in our review suggest that some physicians may value some forms of knowledge more than others. In one of our included studies [27], if a physician thought that mammography for women aged 40 to 49 was not recommended or was unclear, then a patient’s expectation of having mammography tripled the probability that mammography would be ordered for that patient. Our review generally showed that many factors other than clinical guidelines influence physicians in their decision-making with patients, including their colleague’s recommendations [26, 27, 29]. Physicians may at times be as influenced by anecdotal, clinical, and personal experience as they are by evidence generated from conventional sources such as systematic reviews. However, as stated in article 6 of the Code of Ethics of Physicians in Quebec [25], “a physician must practice his profession in accordance with scientific principles”. Moreover, our review did not capture data on system-level factors that may influence the screening perspectives and practices of primary care providers. These factors include quality assurance and performance measurement activities. Mammography screening belongs to the list of performance measures established by the Health Effectiveness Data and Information Set in the United States healthcare system [39].

While this research addresses screening from the perspective of PCPs, it is relevant to consider that in high-income countries, many women are invited to enter mammography screening programs through government-based initiatives. Provincial mammography screening programs across Canada do not offer or advise women to seek counseling prior to entering their programs. In Quebec, at the age of 50, women receive an invitation to enroll in the province’s official screening program. Although the Quebec program offers psychosocial support to women once registered in the program, there is limited access to pre-screening counseling. Once women register in these programs, their physicians typically receive alerts for subsequent mammograms, which are ordered automatically. Few opportunities may exist for women to revisit an initial decision to start screening. For women who discuss screening with their PCP, uncertainty around what information to present to inform decisions remains an important issue. Yet, in the Canadian Medical Association Code of Ethics [17], articles 21 and 22 clearly indicate the ethical obligation of physicians to enable patients in making informed decisions by providing appropriate information and ensuring it is understood. Article 29 in the Code of Ethics of Physicians in Quebec [25] echoes this same responsibility of providers towards their patients.

When balancing the benefits and trade-offs of a screening test becomes less clear, such as in breast cancer screening, primary care experts are increasingly recommending shared decision-making [40]. Throughout this collaborative approach to decision-making, the patient’s personal preferences, values, and beliefs are carefully explored and taken into account. The health provider and patient then deliberate to determine the best option for the patient. Additionally, the patient’s self-efficacy to follow through with a plan and follow-up meetings are critical elements of this decision-making model. Whether or not an actual decision is made, patients’ decisional needs become more evident through this process. Providers and patients can then effectively work together to assess these needs in order to progress in the decision-making [41]. Decision-making support tools such as the SURE Test (Sure of myself, Understand information, Risk-benefit ratio, Encouragement) [42] are useful for practitioners and patients when facing decisional conflict.

Lastly, our review revealed that physicians may have strong intentions to support women in making informed decisions about mammography screening [31], but some physicians may not be engaging in discussions about screening at the time their patients would like [29].

## Limitations

Our study captures articles using diverse methodologies and methods and various outcome measures, resulting in a difficult harmonization of findings. Although all nine included articles are empirical, comparing the results of these studies that measure different outcomes becomes somewhat difficult. Our use of a Critical Interpretive approach [18] allows for a rich set of data that would not have necessarily been included in more rigid search strategies such as those used in systematic reviews. Yet, the conclusions that can be established from our study are perhaps limited and less clear than those that can be made from a systematic review. Our search strategy may have also left out articles relevant to our review, but the McDougall approach [18] seeks to gather key concepts on a topic that emerge from a sub-set of the literature, and we believe our search still resulted in a thorough scanning of relevant literature.

Furthermore, the varying terminology used to describe similar data in our included studies challenged the comparing and contrasting of findings. We were not always able to effectively group data into consistent themes. For instance, in one study, authors measured whether or not physicians ‘offered’ screening [29], whereas in another study [28], the authors measured whether physicians ‘recommended’ screening. We grouped this data together in our analyses, as both indicated a similar disposition towards support for screening for particular patients.

## Conclusions

In conducting this critical interpretive review, we aimed to rigorously gather information on the beliefs and approaches of physicians regarding mammography screening decision-making with average-risk women. As stated in article 3 of the Code of Ethics of Physicians in Quebec [25], physicians must promote and protect the health and well-being of a patient, “both individually and collectively.” This dual responsibility towards both an individual’s needs and to the collective good further emphasizes the need to continue scrutinizing screening.

Upcoming work led by this research group aims to continue this examination, by analyzing comments from physicians in response to clinical evidence on mammography screening. These perspectives, stemming from the Patient Oriented Evidence that Matters (POEMs) dataset, will provide further insight on the decision-making processes occurring during visits with primary care providers and the values guiding the practice of these professionals.

## Abbreviations

AAFP: American Academy of Family Physicians
ACP: American College of Physicians
CTFPHC: Canadian Task Force on Preventive Health Care
PCP: Primary Care Physician
POEMs: Patient Oriented Evidence that Matters
USPSTF: United States Preventive Services Task Force
